# Genome Sequences of 12 Mycobacteriophages Recovered from Archival Stocks in Japan

**DOI:** 10.1128/genomeA.00472-18

**Published:** 2018-06-21

**Authors:** Jumpei Uchiyama, Keijiro Mizukami, Koji Yahara, Shin-ichiro Kato, Hironobu Murakami, Tadahiro Nasukawa, Naoya Ohara, Midori Ogawa, Toshio Yamazaki, Shigenobu Matsuzaki, Masahiro Sakaguchi

**Affiliations:** a School of Veterinary Medicine, Azabu University, Kanagawa, Japan; b Antimicrobial Resistance Research Center, National Institute of Infectious Diseases, Tokyo, Japan; c Kochi University, Kochi, Japan; d Department of Oral Microbiology, Graduate School of Medicine, Dentistry and Pharmaceutical Sciences, Okayama University, Okayama, Japan; e The Advanced Research Center for Oral and Craniofacial Sciences, Dental School, Okayama University, Okayama, Japan; f Department of Microbiology, School of Medicine, University of Occupational and Environmental Health, Kitakyushu City, Japan; g Division of Biosafety Control and Research, National Institute of Infectious Diseases, Tokyo, Japan

## Abstract

Using Mycobacterium smegmatis mc^2^155, 12 siphoviruses were recovered from long-term archival stocks stored in Japan. Their genome sequences were 46.0 to 61.3 kbp with 63 to 68% G+C contents, which allowed them to be categorized within cluster W and subclusters A1, A2, B3, A7, I1, and K4.

## GENOME ANNOUNCEMENT

Mycobacteriophages, which are viruses that infect Mycobacterium spp., have been studied since their first isolation in the 1940s (1). Mycobacteriophages are currently studied as materials for phage therapy and potential bacterial detection for tuberculosis (2, 3).

Using M. smegmatis mc^2^155 as the host, we recently recovered mycobacteriophages from archival stocks stored for approximately 20 to 50 years for application studies (4). Characterization of the recovered 12 mycobacteriophages (C3, BK1, GS4E, D29, A6, B1, PP, Y10, Y2, PR, D12, and HC) showed that they all belong to the family Siphoviridae (4). Here, we report the genome sequences of these 12 recovered mycobacteriophages.

Phage genomic DNA samples were prepared as described previously (4). A single-end library was prepared from each phage genomic DNA sample and was analyzed on a GS Junior 454 sequencer (Roche Diagnostics, Indianapolis, IN, USA). The sequence reads were assembled using 454 Newbler software (version 3.0; 454 Life Sciences, Branford, CT, USA). Gap filling of the contigs was performed by PCR, followed by cycle sequencing. The phage genome sequences were 46.0 to 61.3 kbp long (mean ± standard deviation [SD], 52.7 ± 5.5 kbp), having G+C contents in the range of 63 to 68% (mean ± SD, 65.3 ± 2.1%). Moreover, annotation of the coding sequences (CDSs) was performed using the Microbial Genome Annotation Pipeline (MiGAP) version 2.23 (https://www.migap.org) (5). These genomes contained 78 to 91 CDSs and 0 to 5 tRNAs (mean ± SD, 84.8 ± 5.9 CDSs and 1.3 ± 1.8 tRNAs, respectively).

The Actinobacteriophage Database (PhagesDB) serves as a genome sequence repository for mycobacteriophages (6, 7), which presently categorizes them into genomic clusters and subclusters based on the genomes. The reference data included 1,528 mycobacteriophage genome sequences collected from PhagesDB (downloaded on 20 January 2018) (6). The phylogeny of the recovered phages was analyzed using a phylogenetic analysis tool, ViPTree version 1.0 (8), which computes genome-wide similarity based on the viral genomes by tBLASTx analysis. As a result, phages A6 and BK1 were categorized as subcluster A1; phages GS4E, D29, and C3 were categorized as subcluster A2; phage B1 was categorized as subcluster B3; phage PP was categorized as subcluster A7; phage HC was categorized as subcluster I1; phages Y10 and Y2 were categorized as subcluster K4; and phages PR and D12 were categorized as cluster W. Because of its near-perfect sequence identity match with Mycobacterium virus D29 (GenBank accession number AF022214), the recovered phage D29 was considered to be the clonal strain of Mycobacterium virus D29 isolated in the 1950s (9).

The International Committee on Taxonomy of Viruses (ICTV) classification allowed the taxonomic classification of the recovered phages using these data (10). Phages A6, BK1, GS4E, D29, C3, B1, and PP of cluster A were classified into the genus L5virus. Phage HC of subcluster I1 was classified into the genus Brujitavirus under subfamily Chebruvirinae. Phages Y2 and Y10 of cluster K were classified into the genus Tm4virus. Phages D12 and PR of cluster W could not be classified.

### Accession number(s).

GenBank accession numbers are shown in Table 1.

**TABLE 1:** Genome information for mycobacteriophages recovered from the archival stocks

Phage name	Stock no.^*a*^	Genome length (bp)	G+C content (%)	Coverage (×)	No. of CDSs	No. of tRNAs	GenBank accession no.	Cluster/ subcluster
A6	31	49,728	63.8	61	85	0	AP018478	A1
BK1	63	49,728	63.4	92	85	0	AP018477	A1
C3	39	51,141	63.1	70	88	5	AP018476	A2
D29	76	49,136	63.5	24	79	5	AP018480	A2
GS4E	62	49,823	63.4	77	88	1	AP018479	A2
B1	41	45,961	64.1	56	73	1	AP018485	A3
PP	60	51,509	64.5	51	80	2	AP018486	A7
HC	34	46,305	67.2	42	78	0	AP018487	I1
Y2	25	57,990	67.9	61	91	1	AP018470	K4
Y10	64	57,990	67.9	85	91	1	AP018469	K4
D12	17	61,290	67.6	136	89	0	AP018462	W
PR	37	61,267	67.6	17	90	0	AP018463	W

a Stock number is shown in reference 4.
